# Serological and Genetic Evidence for Altered Complement System Functionality in Systemic Lupus Erythematosus: Findings of the GAPAID Consortium

**DOI:** 10.1371/journal.pone.0150685

**Published:** 2016-03-07

**Authors:** József Prechl, Krisztián Papp, Zoltán Hérincs, Hajna Péterfy, Veronika Lóránd, Zoltán Szittner, Andone Estonba, Paolo Rovero, Ilaria Paolini, Jokin del Amo, Maria Uribarri, Maria Claudia Alcaro, Otsanda Ruiz-Larrañaga, Paola Migliorini, László Czirják

**Affiliations:** 1 Diagnosticum Zrt, Budapest, Hungary; 2 MTA-ELTE Immunology Research Group, Eötvös Loránd University, Budapest, Hungary; 3 Department of Rheumatology and Immunology, Clinic Center, University of Pécs, Pécs, Hungary; 4 Department of Genetics, Physical Anthropology and Animal Physiology, University of the Basque Country, Bilbao, Spain; 5 Department of NeuroFarBa, University of Florence, Florence, Italy; 6 Toscana Biomarkers, Siena, Italy; 7 Progenika Biopharma S.A., a Grifols Company, Derio, Bizkaia, Spain; 8 Department of Clinical and Experimental Medicine, University of Pisa, Pisa, Italy; IMAGINE, FRANCE

## Abstract

Systemic lupus erythematosus is a chronic autoimmune disease with multifactorial ethiopathogenesis. The complement system is involved in both the early and late stages of disease development and organ damage. To better understand autoantibody mediated complement consumption we examined *ex vivo* immune complex formation on autoantigen arrays. We recruited patients with SLE (n = 211), with other systemic autoimmune diseases (n = 65) and non-autoimmune control subjects (n = 149). Standard clinical and laboratory data were collected and serum complement levels were determined. The genotype of SNP rs1143679 in the *ITGAM* gene was also determined. *Ex vivo* formation of immune complexes, with respect to IgM, IgG, complement C4 and C3 binding, was examined using a functional immunoassay on autoantigen microarray comprising nucleic acids, proteins and lipids. Complement consumption of nucleic acids increased upon binding of IgM and IgG even when serum complement levels were decreased due to consumption in SLE patients. A negative correlation between serum complement levels and *ex vivo* complement deposition on nucleic acid autoantigens is demonstrated. On the contrary, complement deposition on tested protein and lipid autoantigens showed positive correlation with C4 levels. Genetic analysis revealed that the non-synonymous variant rs1143679 in complement receptor type 3 is associated with an increased production of anti-dsDNA IgG antibodies. Notwithstanding, homozygous carriers of the previously reported susceptible allele (AA) had lower levels of dsDNA specific IgM among SLE patients. Both the non-synonymous variant rs1143679 and the high ratio of nucleic acid specific IgG/IgM were associated with multiple organ involvement. In summary, secondary complement deficiency in SLE does not impair opsonization of nucleic-acid-containing autoantigens but does affect other antigens and potentially other complement dependent processes. Dysfunction of the receptor recognizing complement opsonized immune complexes promotes the development of class-switched autoantibodies targeting nucleic acids.

## Introduction

Systemic lupus erythematosus (SLE) is a multifactorial chronic autoimmune disease with diverse manifestations. Currently, the disease development is interpreted as a consequence of antinuclear autoantibody production following the breakdown of tolerance due to ineffective clearance of apoptotic debris. The presence of pathological autoantibodies is responsible for decreased complement function and levels, since antibodies and their targets form immune complexes, which consume complement [1]. Antinuclear antibodies, IgG antibodies against double-stranded DNA (dsDNA) and the Sm antigen, antiphospholipid antibodies and impaired function of the classical pathway of complement or decreased serum concentrations of complement C4 or C3 are key markers of the disease [2].

The complement system has been shown to play an intricate role in the development of the disease [3, 4]. Early components of the classical activation pathway play a protective role, while central and terminal components can contribute to disease development. The roles of C1q, the recognition molecule of the classical pathway, in the development of the lupus syndrome can be mapped at the intersection of three key factors: immune complex clearance, adaptive immune response and vascular regeneration [5]. In this triangle complement C1q plays a central role, since it acts as a recognition molecule of apoptotic debris [6], a component of immune complexes [7] and a regulator of endothelial permeability [8]. C1q binding to antigens or antibodies can activate the associated serine proteases C1r and C1s, leading to C2 and C4 cleavage [9]. The activation fragment C4b covalently binds to nearby molecules, molecularly marking the activation site and contributing to the formation of the convertase for C3 cleavage. C3 activation product C3b also covalently binds to the activation site. Using this property of C4 and C3 we have been able to characterize the *in vitro* formation of immune complexes upon the incubation of antigen microarrays with the test serum [10]. Under favorable conditions the binding of antigen specific antibodies to their target antigens is followed by activation of the complement system and on-chip complement deposition. The resulting binding profiles we call functional antibody profiles, because in addition to the binding of antibodies the concomitant deposition of complement products is also recorded.

In our previous study applying functional antibody profiling for the characterization of on-chip immune complex formation in SLE, we showed that in addition to IgG, also IgM, complement C3 and C4 binding to various antigens generates patterns, which are suitable for discriminating healthy serum from SLE serum [11]. Additionally, on-chip complement deposition measurement was more sensitive in identifying SLE patients than IgG binding determination, being able to detect low titer autoantibodies with complement deposition enhancing properties.

Variations in some of the genes encoding complement proteins are associated with SLE. Deficiency of proteins of the early components of the classical pathway, C1q, C4 and C2 strongly predisposes to lupus but is quite infrequent [3]. The *ITGAM* gene codes the CD11b chain of complement receptor 3 and has been shown to be associated with SLE development [12]. The non-synonymous variant rs1143679 (R77H) in this gene has been reported to influence negatively receptor function [13, 14]. This observation may provide biological causative role for this receptor in disease pathogenesis, since impaired uptake by CR3 of complement C4 and C3 opsonized apoptotic debris could lead to the breakdown of tolerance against apoptotic nuclear material[15, 16].

To corroborate and extend these findings we established the GAPAID consortium (Genes And Proteins for AutoImmunity Diagnostics; www.gapaid.eu), a European Union financed project, which was formed with the idea of integrating genetic and serological data in order to improve our understanding of disease pathogenesis and potentially provide novel approaches for the classification of lupus. We employed antigen specific antibody and complement profiling for autoantibody characterization and assessed a polymorphism of the *ITGAM* gene in healthy, non-SLE autoimmune and SLE subjects. In this paper we provide experimental evidence for the imbalance in complement deposition that exists in SLE patient serum, along with genetic aspects of immune complex formation and associations with organ involvement.

## Results

### Clinical and serological characteristics of SLE patients

The basic characteristics of the SLE cohort of PECS and PISA are summarized in Table 1. The two cohorts deriving from a Hungarian and Italian tertiary care center showed very similar clinical-laboratory characteristics. A significant difference was detected regarding disease activity measured by ECLAM (European Consensus Lupus Activity Measurement) scoring [17], renal involvement and complement levels at sampling.

**Table 1:** Clinical and serological characteristics of the SLE patient cohorts.

	PECS cohort n = 146	PISA cohort n = 65	Pooled cohort n = 211
age (years)^a^	45.4±13.7	43.1±14.4	44.7±13.9
women:men	133:13	57:8	190:21
age at onset (years)^b^	30.0 (22.0; 41.0)	27.0 (21.0; 37.5)	29.0 (21.0; 40.0)
disease duration (years) ^a^	13.1±7.6	12.7±10.2	13.0±8.5
ECLAM ^b^	3.0* (2.0; 3.0)	1.0* (0.0; 2.0)	2.5 (1.0; 3.0)
renal involvement	57/146* (39)	49/64* (77)	106/210 (50)
skin involvement	93/146 (64)	43/62 (69)	136/208 (65)
joint involvement	89/146 (61)	38/61 (62)	127/207 (61)
hypocomplementaemia at sampling	55/131* (38)	36/57* (55)	91/188 (43)
anti-dsDNA + at sampling	81/131 (62)	44/64 (69)	125/195 (64)
ANA +	137/145 (94)	57/63 (90)	194/208 (93)
anti-SSA +	65/146 (45)	ND	ND
anti-Smith +	20/144 (14)	ND	ND
anti-cardiolipin IgG +	53/146 (36)	21/56 (38)	74/202 (37)
anti-ß_2_ glycoprotein I +	56/146 (38)	ND	64/181 (35)

Results are presented as ^a^ mean ± standard deviation, ^b^ median (lower quartile; upper quartile), or otherwise as cases/not missing (percentage). Values marked with asterisk are statistically different (p<0.05). ND: not determined or more than 10% missing data; ECLAM: European Consensus Lupus Activity Measurement, ANA: antinuclear antibody

Analysis of functional antibody profiling and *ITGAM* genotyping data was therefore carried out using the pooled cohort.

### IgG and IgM cooperate to trigger complement deposition on nucleic acids in SLE patients

**Fig 1:**
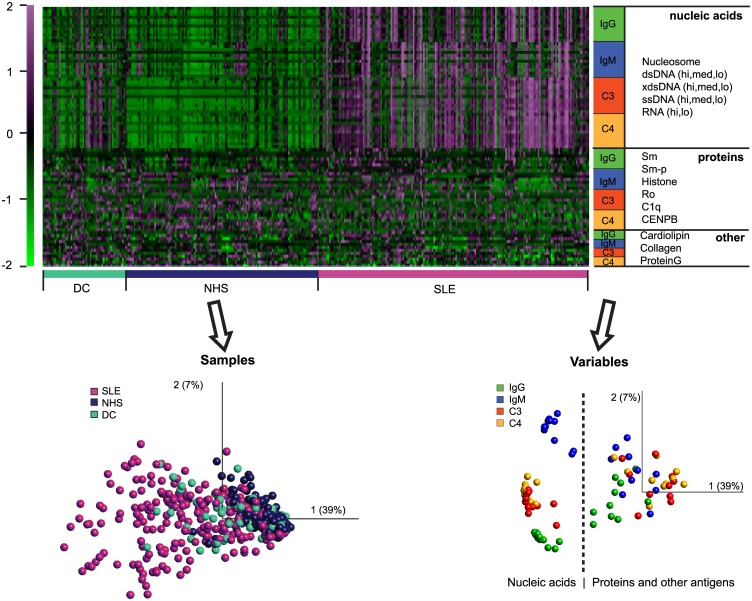
Heatmap and principal component analysis of the functional antibody profiling data. Samples are ordered as study groups. Variables are grouped by the molecular composition and the nature of the detected serum immunoglobulin or complement protein, the order of the particular antigens is shown and applies for each detected protein. PCA plot of samples shows the distribution of samples in the space of the first two components. The variable plot shows the contribution of the variables to the generation of this space, where distance and orientation from the zero origin define a particular interaction’s load in the first two principal components. Percentages in parenthesis indicate contribution of the indicated principal component to overall variability in the dataset. xdsDNA, ultrasound-fragmented dsDNA; CENPB, centromere protein B; Sm-p, peptide of Smith antigen D polypeptide

For the characterization of serological alterations in SLE we compared lupus patients both with non-autoimmune subjects (normal human serum, NHS) and with a diverse collection of patients with different inflammatory rheumatic diseases (disease control, DC), which may be taken into account in the differential diagnosis of SLE. Analysis of antigen microarray binding profiles showed a discriminative pattern on nucleic-acid-containing autoantigens (NA) for IgG, IgM, C3 and C4 in SLE patients, with increased binding of all 4 proteins (Fig 1). The percentage of reactive samples and of positive reactions for serum immunoglobulin and complement proteins in the DC group was intermediate between SLE and NHS samples. Principal component analysis (PCA) of the complete microarray dataset generated two components describing 46% of the overall variation. The first component is mainly responsible for the separation of the study groups, with the second component further subdividing the SLE population. The contribution of IgG and IgM binding to the second component is opposite, as indicated by the position of these variables in the PCA variable space. Importantly, both C3 and C4 binding to NAs shows a pronounced contribution to the first principal component, lying along the axis furthermost from the origin. It is also important to note that some variables are located on the opposite side of the first component; for these variables decreased binding of C4 and C3 to the particular antigens characterizes SLE samples. Because of the strong correlation between C4 and C3 values, from hereon only results of analyses with C4, which showed a somewhat broader distribution and better discriminative properties, are shown.

### Complement C4 deposition measurement is highly sensitive for the presence of autoantibodies against NA

**Fig 2:**
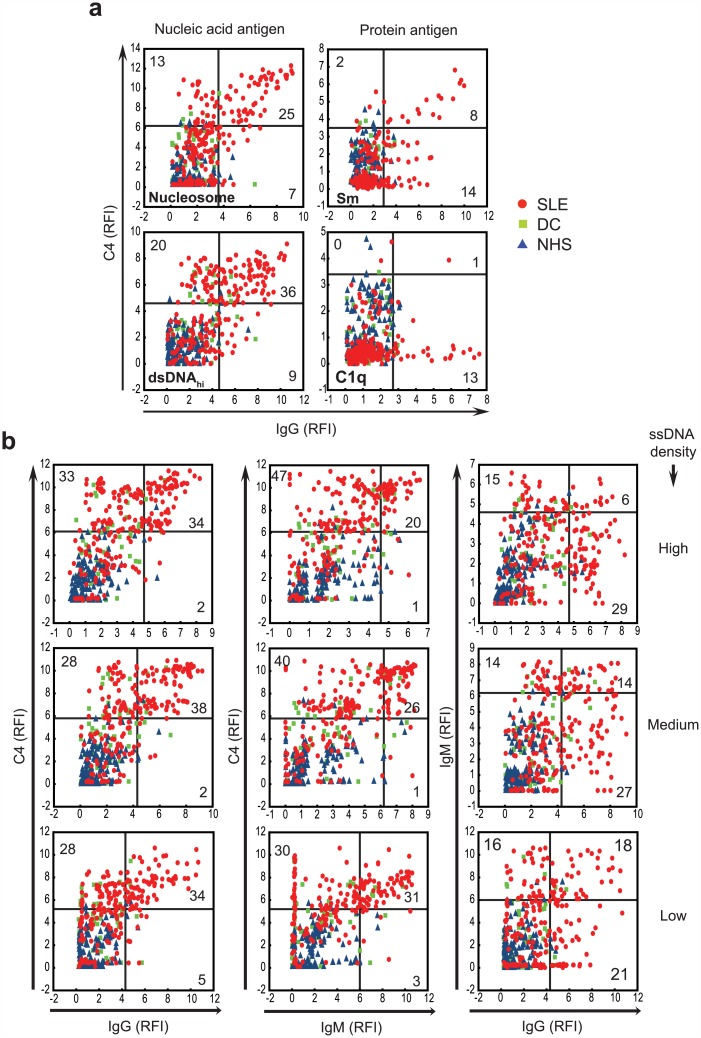
Antibody binding and complement C4 deposition on various antigens. Scatterplots show the relationship between IgG reactivity and C4 deposition in the sera of healthy (NHS), disease control (DC) and SLE subjects. Numbers indicate percentage of SLE subjects in the respective quadrants, which were generated by the 98th percentile boundaries of the NHS group. Antigens of different biochemical nature (a) and of different epitope density (b) are shown. ssDNA, single-stranded DNA

To further examine the relationship between immunoglobulin binding and complement deposition we plotted C4 values against IgG binding (Fig 2a). These plots show that IgG binding to various forms of NAs (see also S1 Fig), results in strong C4 deposition. Even modest or undetectable amounts of IgG triggered C4 deposition in SLE sera. On the contrary, IgG binding did not necessarily trigger C4 deposition on other types of antigens (Fig 2a, S1 Fig).

The cooperation of IgG and IgM in triggering C4 deposition was assessed at various epitope densities of ssDNA (Fig 2b). In SLE patients both IgG and IgM binding was observed even at the lowest epitope density, some samples showing IgG, others IgM dominance. For each variable we adjusted a cut-off value that allowed 2 percent of the normal, non-autoimmune subjects to be misqualified as positive. Using this 98% specificity threshold, the sensitivity of identifying SLE samples was the highest by C4 deposition detection (sum of values in upper quadrants of first two columns: 67%, 66% and 61%) at all three epitope densities tested, exceeding also the combined use of IgG with IgM (sum of values in upper and lower right quadrants of right column: 50%, 55% and 55%, respectively) (Fig 2b).

### Complement deposition on NA is robust in spite of decreased serum complement levels

**Fig 3:**
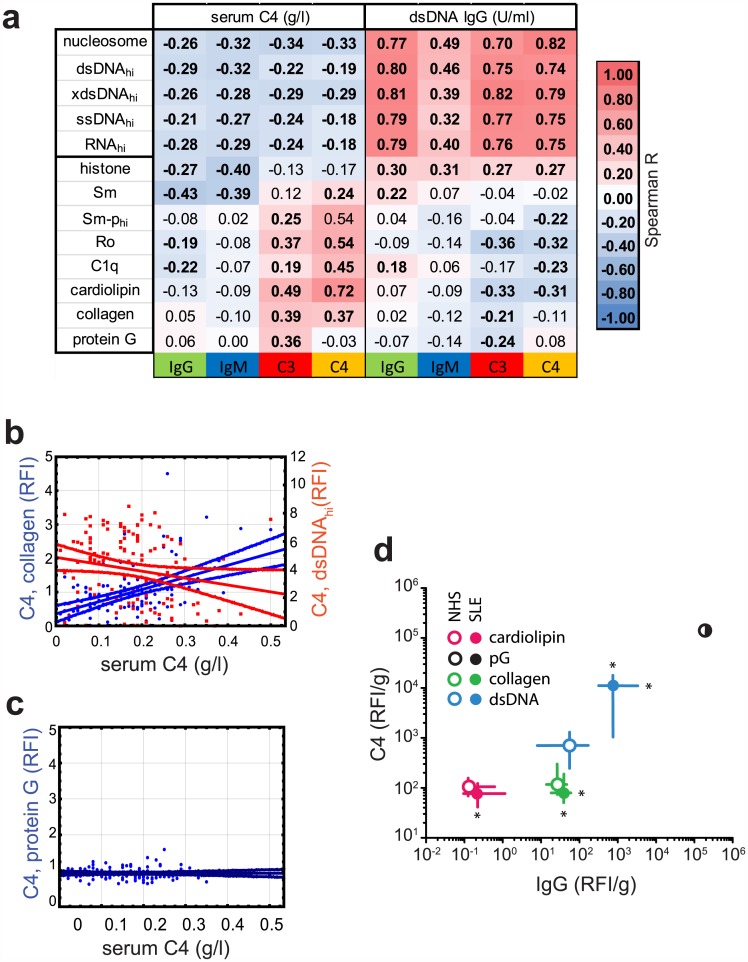
Opposite complement activating properties of biochemically different antigens in SLE. The PECS SLE cohort with available serum complement concentrations (n = 124) was analyzed for associations between serum protein levels and on-chip antigen binding of immunoglobulin and complement proteins. **a** Spearman rank-correlations of serum C4 concentrations, dsDNA IgG reactivity with various microarray derived binding data. Values indicate r, bold fonts highlight significant correlations (p<0.05). **b** Comparative C4 deposition on dsDNA and collagen as a function of serum C4 concentrations. Linear regression fits with 95% confidence intervals are shown. **c** Complement C4 deposition initiated by superantigen protein G as a function of serum C4 concentration. **d** Comparative C4 deposition and IgG binding on various antigens on a mass basis in healthy and SLE serum samples. Asterisks indicate direction and significance (p<0.05) of changes in the SLE versus NHS group. pG, Staphylococcal protein G.

A potential risk factor of using C4 deposition measurement for the serological diagnosis of SLE could be the secondary complement deficiency due to complement consumption by immune complexes. Therefore we compared serum complement levels and dsDNA specific IgG levels with various microarray-derived variables in a group of SLE patients (Fig 3a, 3b and 3c). The binding of IgG and IgM to NAs was negatively correlated with serum C4 levels (Fig 3a). However, on-chip C3 and C4 deposition to NAs was also negatively correlated with serum C4 levels. Strong C4 deposition on dsDNA was observed even at C4 levels below the normal range (<0.1 g/l) (Fig 3b). Unlike NAs, binding of C4 and C3 to other antigens showed positive correlation with serum levels of C4. To establish whether IgG initiated early complement activation events were generally impaired we looked at C4 deposition on a bacterial superantigen, protein G (Fig 3c), finding no influence of serum C4 concentrations.

In order to estimate the capacity of these autoantigens to accept covalent complement binding we generated a graph using raw binding data on an antigen mass basis, which is suitable for inter-antigen comparisons (Fig 3d). dsDNA was about tenfold better C4 acceptor compared to collagen and cardiolipin when incubated in NHS serum. Upon incubation in SLE serum this difference further increased, along with increased IgG binding, resulting in higher than 100-fold differences.

### The rs1143679 polymorphism in ITGAM affects antinuclear antibody production

**Fig 4:**
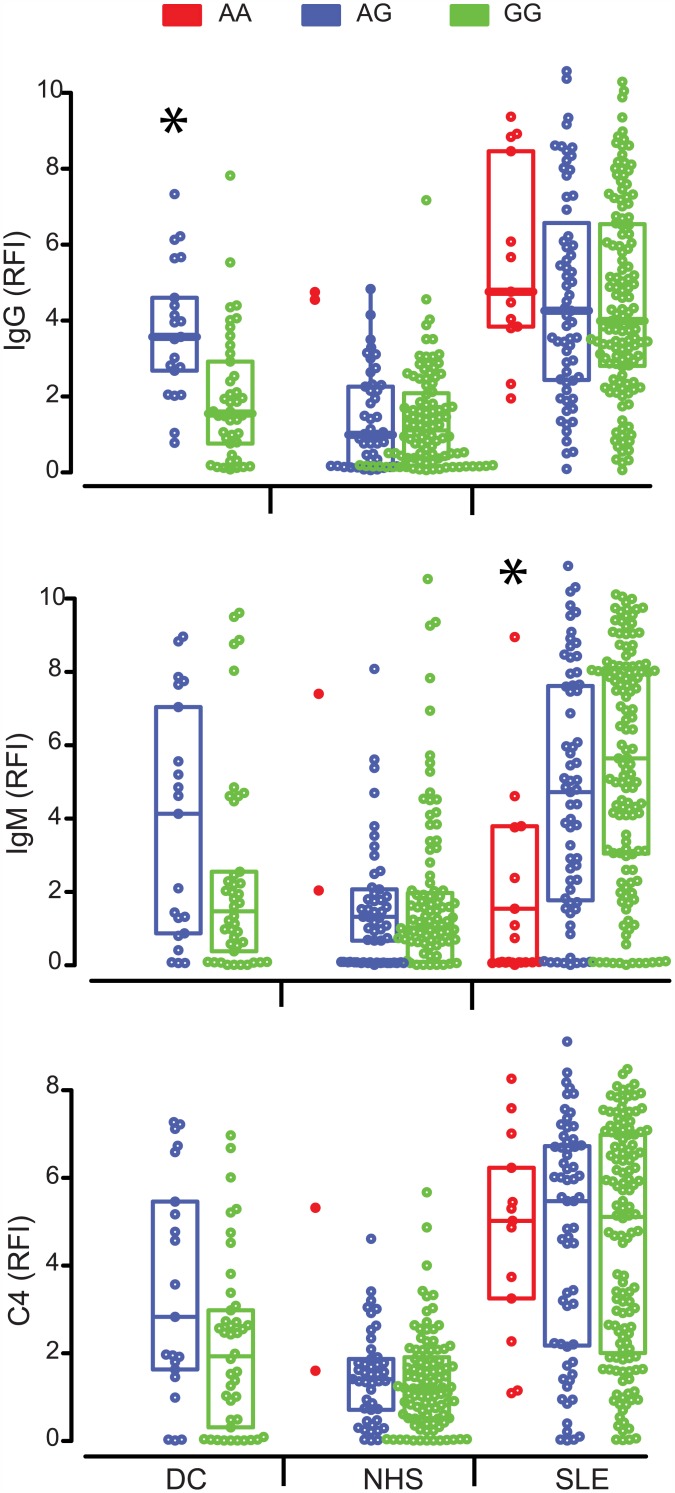
Effect of SNP rs1143679 genotype on dsDNA specific antibody levels and C4 deposition. Binding of IgG, IgM and C4 to dsDNA was determined by functional antibody profiling analysis. Individuals within the study groups were classified based on their genotype. Boxes show interquartile ranges, horizontal lines stand for median. Asterisks indicate statistically significant (p<0.05) difference from the GG genotype. AA genotype carriers in the NHS group were excluded because of the low number of samples.

Antigens composed of NAs are thus decorated with complement C4 even in healthy persons, this deposition is further increased in the presence of antibodies against NA. The cleaved form of C4, C4b is a ligand of complement receptor 3, which is expressed on phagocytic cells. We next looked at the effect on dsDNA specific antibody levels and complement deposition of SNP rs1143679 in the *ITGAM* gene, which encodes a subunit of complement receptor 3 (CR3) (Fig 4). A genotypic effect was observed in the DC group, with carriers of AG genotype showing significantly increased dsDNA reactive IgG levels compared with carriers of GG genotype. IgG binding to dsDNA was also higher in homozygous A carriers of the NHS group than in the other two genotypes but only two samples belonged to this subgroup. A similar but non-significant effect was observed for IgM binding to dsDNA in the DC and NHS groups. Surprisingly, a significantly lower IgM binding was observed for AA genotype carriers versus the GG genotype in the SLE group. This opposite effect of AA genotype on antibody production in the SLE group canceled out when C4 deposition was measured (Fig 4).

### Association of the composition of on-chip immune complexes with organ involvement

**Fig 5:**
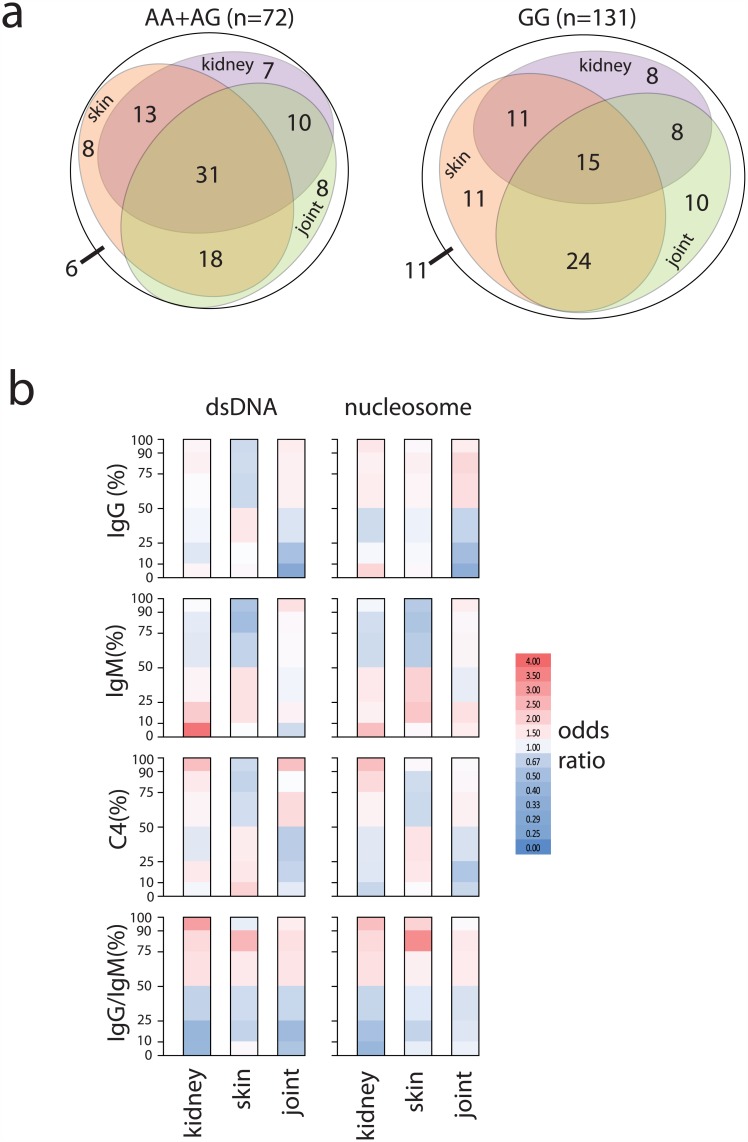
Association of organ involvement with ITGAM genotype and immune complex composition. **a**. Percentage of the SLE patients with the involvement of the indicated three organs is shown for rs1143679 genotypes. The presence of single, double or triple organ involvement is illustrated by Euler ellipses with areas proportional to the percentages shown. **b**. Association with organ involvement of the level and composition (IgG, IgM, C4 and IgG/IgM) of immune complexes formed on dsDNA and nucleosome is indicated. Odds ratios for patients falling into the indicated percentile groups (upper 10^th^, 25^th^, 50^th^ and lower 10^th^, 25^th^, 50^th^ percentiles) for the given measurements are shown. Positive odds (>1) are in red, negative odds (<1) are in blue.

Deposition of immune complexes in various tissues leads to inflammation and consequent organ damage in SLE. We were interested whether the complement content of the on-chip generated complexes showed any association with organ involvement. First we looked at overlaps between kidney, joint and skin involvement in SLE patients carrying and lacking the rs1143679 susceptibility allele A (Fig 5a). Most of the patients had at least two of these organs affected by the disease, with significantly higher percentage of kidney involvement in the A allele carriers (Chi-square test, p<0.05).

We analyzed on-chip generated immune complex composition of two NA antigens of special importance in SLE diagnostics, dsDNA and nucleosome. Odds ratios of the presence of organ involvement for various percentile ranges of the indicated binding proteins were calculated (Fig 5b). The binding of IgG, IgM and C4 to both autoantigens showed markedly different patterns of organ associations. Weak binding of IgG was negatively associated with joint involvement. Low levels of dsDNA and nucleosome specific IgM were associated with kidney involvement. Strong C4 deposition on dsDNA was positively associated with kidney and joint involvement. The ratio of IgG/IgM binding showed similar odds patterns for both antigens and all three organs. (Fig 5b).

## Discussion

While specific autoantibody testing has become a cornerstone for SLE diagnosis, the biological and molecular events affecting the complement system in SLE are still measured indirectly, by assessing complement consumption. This approach has pitfalls inherent to the high individual variability of complement levels. Only recently have some groups proposed the testing for C4 fragments deposited *in vivo* on blood corpuscles [18], such as red blood cells, thrombocytes and lymphocytes. We worked out a different technology, where *ex vivo*, on-chip complement deposition is measured in an antigen specific manner. Using this technology we showed earlier that complement C4 and C3 deposition patterns are suitable additional tools for SLE diagnostics [11]. The data presented in this paper validate and extend those results.

Autoantigens made of or containing NA, such as dsDNA, ssDNA, RNA and nucleosome, show a behavior that is strikingly different from other types of antigens. This is demonstrated by the increased binding of all four tested proteins (IgM, IgG, C4, C3) to these antigens from SLE sera (Fig 1), the differential effects of IgG binding on C4 deposition (Fig 2) and the inverse correlations with serum complement and dsDNA antibodies (Fig 3a and 3b). This intricate relationship between the complement system, lupus and nucleic acids is further underlined by the fact that carriers of the susceptible genotype of *ITGAM* show altered antibody responses to NA but not other SLE-related autoantigens (Fig 4 and not shown). Additionally, NAs have a remarkably strong capacity to bind complement products as compared to antigens with different biochemical properties (Fig 3d). This is partly attributable to direct binding of C1q to DNA [19] and also to indirect binding mediated by other NA recognition molecules, such as IgM [20] and CRP [21, 22], which in turn also bind C1q. Of note, ssDNA specific IgM showed quantitative and qualitative differences in the NHS and SLE groups: high avidity binding and stronger C4 activation was observed in the latter (Fig 2b). Since both IgM and IgG can trigger complement deposition, the arrangement of C3 and C4 binding to NAs in the PCA plot (Fig 1c) reflects the cooperative contribution of these immunoglobulin isotypes to complement binding and suggest that complement deposition measurements have the highest discriminative sensitivity. This is confirmed by the observed relationship between IgG, IgM and C4 binding (Fig 2).

Assuming that on-chip IgG and IgM binding and complement deposition reflects in vivo events we interpret these results as a skewed complement consumption in peripheral blood. In spite of decreased complement protein levels and decreased C4 deposition on non-NA antigens there is massive C4 deposition on NAs. This suggests that the early classical pathway components are very actively extracted from the blood by NAs and a functional deficiency exists for all other processes, which require the activity of this pathway. The initiator molecule of classical pathway complement, C1q is known to have several binding partners ([23]). Recent observations suggest that it plays a role in vascular endothelium maintenance ([8]), so its relative deficiency could interfere with vascular regeneration, thereby contributing to lupus pathogenesis ([5]).

**Fig 6:**
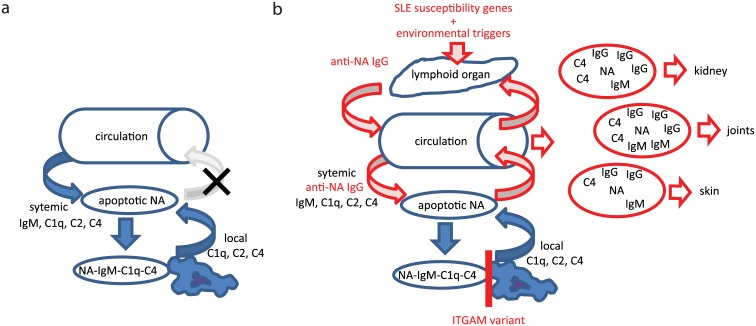
Scheme for the role of complement in the pathogenesis of anti-dsDNA IgG positive SLE. **a**. Apoptotic DNA is normally quickly opsonized by systemic IgM, C1q and early complement activation products C4 and C2, locally produced opsonins also helping removal. This opsonized debris is swiftly removed by tissue macrophages, thus the level of debris is controlled in the circulation and other organs are protected from its pathological accumulation. **b**. Inefficient local and systemic removal by dysfunctional CR3 encoded by *ITGAM* on tissue and sinusoidal macrophages results in increased levels of opsonized debris in the circulation. Circulating immune complexes reach lymphoid organs and trigger production of antigen specific antibodies. In the presence of other SLE susceptibility genes and environmental triggers high avidity DNA specific IgG develops. Upon reaching the circulation this IgG enhances complement consumption by apoptotic DNA and triggers FcgammaR mediated inflammation in tissues where immune complexes are deposited. Relative IgG, IgM and C4 content of immune complexes may influence tissue deposition preference.

Apoptotic cellular debris displaying NAs thus consumes complement powerfully ensuring complement opsonization (Figs 3 and 6). These apoptotic vesicles, nucleosomes and complexes covered with complement C3b and C4b are removed from the circulation by various cells and receptors, including CR3. CR3 is an integrin composed of two chains, one of them encoded by the *ITGAM* gene. Altered function of this receptor has been proposed to be responsible for the development of anti-dsDNA antibodies, since another SNP (rs9888739) in *ITGAM* has been shown to be associated with anti-dsDNA positive SLE rather than anti-dsDNA negative SLE [24]. This is supported by our observation that carriers of the susceptibility allele showed stronger anti-dsDNA IgM and IgG reactivity in the DC group (Fig 4). While there was a tendency of increased anti-dsDNA IgG reactivity in homozygous susceptibility allele carriers of the SLE group, a significant genotypic effect was only observed in IgM reactivity. Interestingly, this was a decrease rather than an increase in reactivity. We suspect that other susceptibility genes and environmental factors responsible for SLE development promote isotype switching in the susceptibility allele carriers to an extent that the majority of dsDNA specific cells switch from IgM to IgG production. The ratio of dsDNA specific IgG and IgM has been reported to be predictive of lupus nephritis development [25]. Since the susceptibility allele (A) at rs1143679 in *ITGAM* is also associated with lupus nephritis development [26], our observations of increased anti-dsDNA IgG/IgM ratio in this group (S2 Fig) provide a causative relationship between genotype and phenotype.

To identify associations between organ involvement and the composition of on-chip formed immune complexes we calculated the odds of being effected for three organs in different percentile groups of IgG, IgM and C4 reactivity (Fig 5). Our results confirm that anti-dsDNA IgM is negatively associated with kidney involvement [27]: SLE patients in the lowest 10-percentile group had higher odds of renal disease. While high avidity dsDNA specific IgG is thought to play a role in SLE pathogenesis and shows association with disease activity [28] in our hands only kidney and joint involvement was mildly associated with anti-dsDNA IgG levels. Though the presence of the *ITGAM* susceptibility allele was associated with increased frequency of kidney involvement, in fact the increase affected not single kidney but rather combined involvement of the kidney (Fig 5). Accordingly, the ratio of IgG/IgM binding showed similar association with all three organs. Thus, increased NA specific IgG/IgM ratio due to ITGAM polymorphism increases susceptibility to kidney involvement together with skin and joint involvement (Fig 6).

To conclude, our *ex vivo* antibody and complement binding assays highlight that nucleic acids consume complement with very high efficiency, a phenomenon further enhanced by antibodies recognizing these antigens (Fig 6). A dysfunction in immune complex removal caused by mutations in *ITGAM* promotes the development of NA specific antibodies, which is a last resource for achieving NA debris clearance by the organism. Unfortunately these IgG containing particles and complexes get trapped at certain sites of the body, triggering inflammation via Fc gamma receptors. The binding of the complexes to the vessel wall and the extracellular matrix is very likely mediated by the recognition molecule of the classical complement pathway C1q [7], targeting leaky endothelium and fenestrated endothelium by nature of its affinity for anionic ligands [5]. The physicochemical and immunological nature of these immune complexes may determine where the complexes are trapped, therefore the immunological composition is associated with organ involvement.

## Materials and Methods

### Patient recruitment and sampling

211 consecutive SLE patients (SLE) classified according to the 2012 Systemic Lupus International Collaborating Clinics classification criteria [2] were recruited between 2012 September and 2013 July from the University of Pécs (PECS), Medical Center, Department of Rheumatology and Immunology and from the University of Pisa (PISA), respectively. Besides control samples (NHS) derived from healthy volunteers (n = 149), serum samples from patients with different connective tissue diseases (n = 65) were also used as a second set, termed disease control (DC) samples: Sjögren’s-syndrome [29] (n = 13), systemic sclerosis [30] (n = 41) and undifferentiated connective tissue disease (n = 10), psoriatic arthritis (n = 1).

All patients gave their written informed consent to the study. The relevant ethics committees of Hungary (Egészségügyi Tudományos Tanács, Tudományos és Kutatásetikai Bizottság) and Italy (Comitato Etico Area Vasta Nordovest, Azienda Ospedaliera Universitaria Pisana, Pisa) gave their approval for conducting study with the following contract numbers, respectively: 24973-1/2012/EKU (658/PI/2012.), 45066/2012. All procedures followed were in accordance with the ethical standards of the responsible committee on human experimentation and with the Helsinki declaration of 1975, as revised in 2008.

Blood samples were collected according to standard procedure; the buffy coat layer and serum were collected and stored at –20°C for further processing.

### Clinical characteristics of patients

Data on clinical manifestations (kidney, joint and skin involvement) and basic serological characteristics were obtained from the patient charts. The disease activity of SLE patients was assessed by the European Consensus Lupus Activity Measurement (ECLAM) score [17].

Hypocomplementemia was defined as low complement 3 and/or complement 4 levels as determined by nephelometry. dsDNA-IgG ELISA measurement was carried out by Aeskulisa dsDNA-G ELISA kit following the instructions of the manufacturer.

### Genotyping of SNP rs1143679 from ITGAM gene

DNA from the buffy coat of all collected samples was purified by NucleoSpin 96 Blood Core Kit (Macherey-Nagel). DNA quantity (ng/ul) and quality (260/280 and 260/230 absorbance ratios) were checked with Qubit fluorometer and NanoDrop 8000 Spectrophotometer, respectively. Genotyping of Single Nucleotide Polymorphism (SNP) rs1143679 (R77H) from *ITGAM* gene was performed by BioMark^™^ HD System (Fluidigm) based on the 5’ exonuclease activity of the polymerase. For each array, 2 negative controls and 46 samples were included. Fluidigm SNP Genotyping Analysis Software v.3 was used for allele assignation. Prior to statistical analyses two quality criteria were checked with PLINK v.2.050 software [31]: SNP call rate, which reached the 99.5% (min. 95%), and conformity of genotype proportions to Hardy-Weinberg equilibrium (HWE) for the SNP in the overall population (*P* value > 0.05).

### Microarray measurements

25 different antigens and controls (antigens listed in GEO database) were printed onto 16-pad nitrocellulose-covered FAST slides (Main Manufacturing) by sciFlexarrayer S11 (Scienion AG, sciArraying service). Different dilutions of the antigens were printed in triplicates then stored at 4°C in sealed bags. Dried arrays were rinsed in PBS for 15 minutes before use, then incubated with diluted serum at 37°C for 1 hour. Two separate measurements were applied on each sample for detection of bound components. Five-fold serum dilution in 2.5mM Ca2+, 0.7mM Mg2+ and 5% BSA complemented PBS buffer was used for detection of deposited C3 and C4 complement fragments. 125-fold serum dilution in 25mM EDTA, 0.05% Tween 20 and 5% BSA complemented PBS buffer was applied for measurement of bound antigen specific IgG and IgM antibodies. Serum treated slides were washed with PBS containing 0.05% Tween 20, then incubated in the mixture of 1:5,000 diluted FITC-conjugated F(ab’)2 fragment of goat anti-human C3 antibody (Cappel) and 1:1000 diluted Alexa 647 conjugated goat anti-human C4 (Cappel, in-house conjugation) or 1:2500 diluted DyLight 488-conjugated F(ab’)2 fragment goat anti-human IgM (mu chain specific) (Jackson ImmunoResearch) and 1:2,500 diluted DyLight 649-conjugated F(ab’)2 fragment goat anti-human IgG (gamma chain specific) (Jackson ImmunoResearch). Labeling with fluorescent antibodies was carried out at room temperature for 30 minutes in PBS containing 5% BSA and 0.05% Tween 20. After washing in PBS containing 0.05% Tween 20, arrays were dried and scanned by FLAIR Fluorescent Array Imaging Reader (Sensovation).

### Analysis of microarray data

Data were analyzed by Array-Reader software by Sensovation, Radolfzell, Germany. Signal intensities were calculated by subtracting local background from medians of the three parallel signal intensities. Negative signals values were clamped to arbitrary value 1. Normalization procedure was carried out in two steps. First, slide to slide normalization was applied separately to the two print-batches of microarray slides. Slide specific normalization factors were calculated based on the signals in the subarray that was treated only with buffer. Human IgM, human IgG, Staphylococcal protein G (pG) and human C4 features on arrays were used for normalization of IgM, IgG, C3 and C4 signals, respectively. This adjustment compensated for both overall biological variations in the samples and technical differences in the detection. A second normalization step was applied to compensate the possible differences in the two printing batches. Values in a given batch were divided by geometric means of values derived from the given antigen in control serum samples. These values were increased by one and base 2 logarithm was calculated. The data discussed in in this publication have been deposited in the National Center for Biotechnology Information’s Gene Expression Omnibus (GEO) [32] and are accessible through GEO series accession number GSE69372.

### Analytical and statistical methods

Normality of clinical variables was tested with Shapiro-Wilk test. Since none of the variables were found to have standard normal distribution, the SLE-cohort of PECS and PISA were compared using Mann-Whitney U-Test and Fisher’s exact test. Principal component analysis (PCA) was carried out using Qlucore software (Qlucore AB, Lund, Sweden). Spearman correlation coefficients Chi-square statistics were calculated using Statistica software from Statsoft. Mann-Whitney U-Test was used for comparing IgG binding and C4 deposition in the NHS and SLE groups on various antigens. For the statistical analysis of rs1143679 (*ITGAM*) genotype effects Kruskal-Wallis test was used for the SLE group where the three genotypes were present, while Mann-Whitney test was used for comparing GG to GA genotypes in the DC and NHS groups where the AA genotype was absent or negligible. Euler ellipses were calculated and drawn with eulerAPE 3.0.0 software [33].

## Supporting Information

S1 Fig Complement C4 deposition on nucleic-acid-containing and protein antigens. Scatterplots show the relationship between IgG reactivity and C4 fixation in the sera of healthy (blue), DC (green) and SLE (red) subjects. Numbers indicate percentage of SLE subjects in the respective quadrants, which were generated by 98th percentile boundaries of the NHS group. xdsDNA, ultrasound-fragmented dsDNA; Sm-p, peptide of Smith antigen D polypeptide
(PDF)

S2 Fig Effect of SNP rs1143679 genotype on dsDNA specific antibody level ratios. Binding of IgG and IgM to dsDNA was determined by functional antibody profiling analysis. Individuals within the study groups were classified based on their genotype. Boxes show interquartile ranges, horizontal lines stand for median. Asterisks indicate statistically significant differences between groups linked by horizontal lines; * p<0.05, *** p<0.001, Mann-Whitney U test. AA genotype carriers in the NHS group were excluded from analysis because of the low number of samples.
(PDF)
