# Supplementary figures and images for: Serological and Genetic Evidence for Altered Complement System Functionality in Systemic Lupus Erythematosus: Findings of the GAPAID Consortium
Source: PLoS One. 2016 Mar 7;11(3):e0150685. doi: 10.1371/journal.pone.0150685 (PMC4780778; doi:10.1371/journal.pone.0150685)

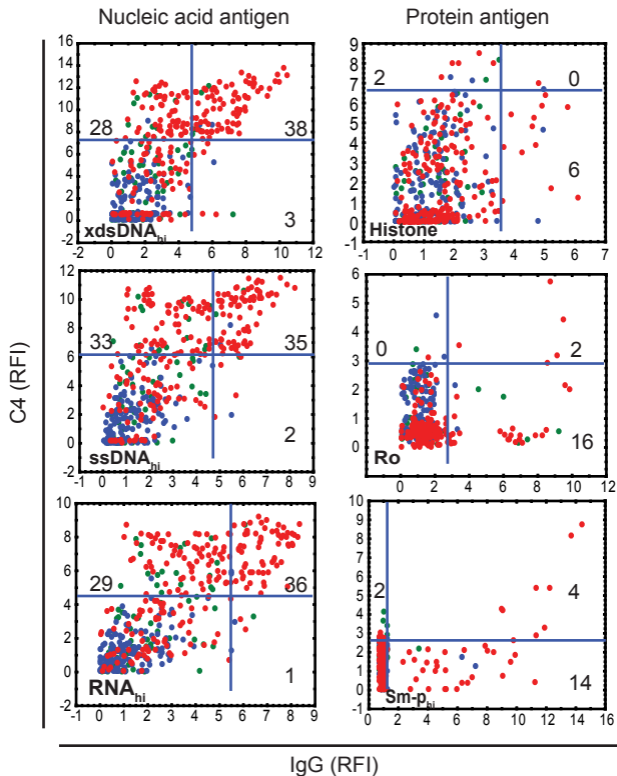

**Supplementary Figure 1.**

Supplement: S1 Fig — Scatterplots show the relationship between IgG reactivity and C4 fixation in the sera of healthy (blue), DC (green) and SLE (red) subjects. Numbers indicate percentage of SLE subjects in the respective quadrants, which were generated by 98th percentile boundaries of the NHS group. xdsDNA, ultrasound-fragmented dsDNA; Sm-p, peptide of Smith antigen D polypeptide (PDF) [file pone.0150685.s001.pdf]

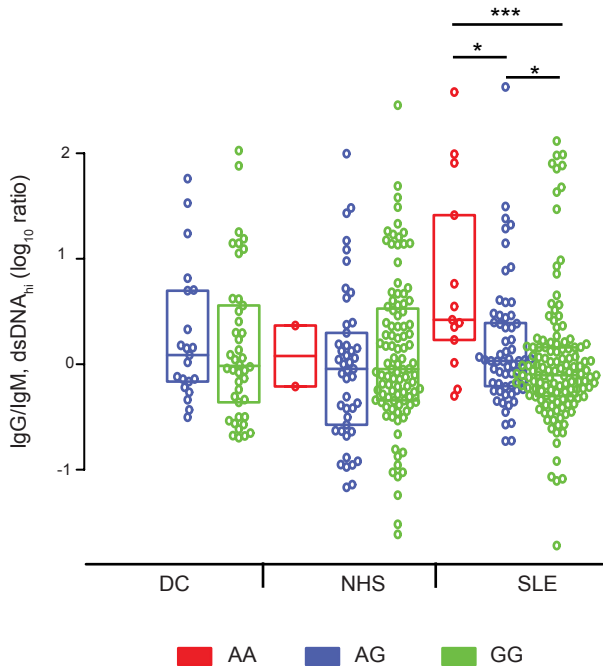

Supplement: S2 Fig — Binding of IgG and IgM to dsDNA was determined by functional antibody profiling analysis. Individuals within the study groups were classified based on their genotype. Boxes show interquartile ranges, horizontal lines stand for median. Asterisks indicate statistically significant differences between groups linked by horizontal lines; * p<0.05, *** p<0.001, Mann-Whitney U test. AA genotype carriers in the NHS group were excluded from analysis because of the low number of samples. (PDF) [file pone.0150685.s002.pdf]
